# 

**Published:** 1907-10-05

**Authors:** 


					i ,n: r; ; rrr
I <K?Wvi*f OU '?? r '?r. ?/ 0* f~
OCT, I K nn,7 ,f- ~j <^r
^SPITALr
^ Telegraph*^" Address: THE MODERN
\f ?- jl* ""'Telephone Number :
ii.d.u, London. MEDICAL JOURNAL. V^%, G<rra'd'
si ?IL  f 'i
MEW SERIES. ' No. 28, Vol. II. I S atttrti AV
No. 1100. Vol. XLIII. DA1UK1JA*.
October 5tii, 1907.

				

